# Interspecies interactions and potential Influenza A virus risk in small swine farms in Peru

**DOI:** 10.1186/1471-2334-12-58

**Published:** 2012-03-15

**Authors:** Sarah McCune, Carmen S Arriola, Robert H Gilman, Martín A Romero, Viterbo Ayvar, Vitaliano A Cama, Joel M Montgomery, Armando E Gonzales, Angela M Bayer

**Affiliations:** 1 Asociación Benéfica Proyectos en Informática, Salud, Medicina y Agricultura (AB PRISMA), Lima, Peru; 2 Facultad de Medicina Veterinaria, Universidad Nacional Mayor de San Marcos, Lima, Peru; 3 Department of International Health, Johns Hopkins Bloomberg School of Public Health, Baltimore, MD, USA; 4 Facultad de Salud Pública y Administración, Universidad Peruana Cayetano Heredia, Lima, Peru; 5 Center for Global Health, Universidad Peruana Cayetano Heredia, Tumbes, Peru; 6 Centers for Disease Control and Prevention, Atlanta, GA, USA; 7 Naval Medical Research Unit - 6, Lima, Peru; 8 Division of Infectious Diseases, David Geffen School of Medicine, University of California, Los Angeles, Los Angeles, CA, USA

## Abstract

**Background:**

The recent avian influenza epidemic in Asia and the H1N1 pandemic demonstrated that influenza A viruses pose a threat to global public health. The animal origins of the viruses confirmed the potential for interspecies transmission. Swine are hypothesized to be prime "mixing vessels" due to the dual receptivity of their trachea to human and avian strains. Additionally, avian and human influenza viruses have previously been isolated in swine. Therefore, understanding interspecies contact on smallholder swine farms and its potential role in the transmission of pathogens such as influenza virus is very important.

**Methods:**

This qualitative study aimed to determine swine-associated interspecies contacts in two coastal areas of Peru. Direct observations were conducted at both small-scale confined and low-investment swine farms (n = 36) and in open areas where swine freely range during the day (n = 4). Interviews were also conducted with key stakeholders in swine farming.

**Results:**

In both locations, the intermingling of swine and domestic birds was common. An unexpected contact with avian species was that swine were fed poultry mortality in 6/20 of the farms in Chancay. Human-swine contacts were common, with a higher frequency on the confined farms. Mixed farming of swine with chickens or ducks was observed in 36% of all farms. Human-avian interactions were less frequent overall. Use of adequate biosecurity and hygiene practices by farmers was suboptimal at both locations.

**Conclusions:**

Close human-animal interaction, frequent interspecies contacts and suboptimal biosecurity and hygiene practices pose significant risks of interspecies influenza virus transmission. Farmers in small-scale swine production systems constitute a high-risk population and need to be recognized as key in preventing interspecies pathogen transfer. A two-pronged prevention approach, which offers educational activities for swine farmers about sound hygiene and biosecurity practices and guidelines and education for poultry farmers about alternative approaches for processing poultry mortality, is recommended. Virological and serological surveillance for influenza viruses will also be critical for these human and animal populations.

## Background

The recent epidemic of highly pathogenic avian influenza (HPAI) in Asia and the 2009 pandemic of so-called "Swine flu" (H1N1) demonstrated that influenza A viruses (IAVs) continue to pose a critical threat to global public health. The animal origin of the viruses highlighted the potential for interspecies transmission that may lead to viral reassortment. Thus, it is important to examine current livestock-raising activities and biosecurity practices in areas where small-scale farming is frequent and critical for local livelihoods.

Wild, aquatic birds-especially ducks and geese-are widely recognized to be reservoirs of IAVs [1]. Numerous avian influenza strains have been isolated from migratory and nonmigratory waterfowl in Peru and in other South American countries [2]. The transmission route of IAVs in aquatic birds is fecal-oral, and feces can contain highly concentrated amounts of the virus [3,4]. Indeed, a gram of virus-infected feces can contain up to ten billion viral particles [5]. Moreover, IAVs can survive in water for several days [6] allowing them to be transmitted among waterfowl at close range and through avian migration over significant distances [4,7]. Evidence from Asia shows that avian influenza viruses are also able to be transmitted to and infect a variety of mammals. There are several examples of the infection of dogs by H3N2 [8-10], an example from Cambodia of the probable infection of five cat species by H5N1 [11], and isolation of the H5N2 virus in pigs in Korea [12].

Interspecies infection can occur by jumping or adaptation of an entire virus to a new host species or through the process of reassortment, during which viruses from different species mix to create a new virus that may be more transmissible or pathogenic. Domestic birds and mammals-including humans, swine, horses, ferrets, cats, mink, and even seals and whales-are all susceptible to infection by reassortment IAVs [3,13-16]. Though it has never been confirmed *in vivo*, swine, in particular, are generally hypothesized to be prime "mixing vessels" because their trachea has receptors for both avian and human influenza viruses [3,17-20]. Castrucci et al. (1993) observed reassortment of avian and human viruses in Italian swine [21], and an H3N2 virus that was isolated in American swine contained a combination of avian and human influenza strains [22,23].

Increased human consumption of meat, more efficient animal husbandry practices and the resulting profit potential have led an increased number of farmers in the developing world to forego traditional practices, such as free-range or grazing, in favor of small-scale intensive models. The Council for Agricultural Science and Technology (1999) notes that these more intensive farms are replacing traditional models at a rate of 4.3 percent per year, especially in South America, Africa, and Asia, with poultry and swine farms outpacing any other livestock subsector [24]. Given these changing dynamics of livestock production, this study used qualitative methods to better understand current interspecies interactions and biosecurity practices on small-scale confined and low-investment swine farms in two distinct coastal communities of Peru.

## Methods

### Ethics statement

The Universidad Peruana Cayetano Heredia ethical review committee, which has Federalwide Assurance (FWA), approved the research protocol and consent process. All participants provided informed verbal consent prior to data collection and to any audio recording. Verbal consent was considered appropriate for this study since many of the farms are not approved by law. Therefore, written consent could link the participant's name and signature to participation in a study about illegal farming and place the person at risk. Instead, the member of the research team obtained each participant's verbal consent and then the team member wrote and signed his/her name to document the process. No further permissions to observe the farms were necessary since all of the farms are small and the person providing consent was also the farm owner.

### Study settings

This study was carried out in two coastal sites in Peru: Chancay and Tumbes.

Chancay, located 83 kilometers north of Lima on the central coast of Peru, is a semi-urban district with a population of 49,932. This small city is composed of *centros poblados*, or population centers akin to neighborhoods. The site of this investigation was one such *centro poblado*, a community of approximately 250 small- to medium-sized swine farmers. The boundaries of the study site are the Pacific Ocean to the west, large sand dunes and agricultural fields to the east, the commercial center of Chancay to the north, and a protected avian wetland sanctuary to the south. Swine farming in Chancay is considered to be "small-scale, confined pig production" [25]. The swine are corralled, meaning that they do not run freely or scavenge, and are entirely dependent on humans for food and water.

Tumbes, located in the northernmost coastal region of Peru, is bordered by Ecuador to the North. The city of Tumbes, the regional and provincial capital, is home to 94,702 inhabitants. Tumbes is a largely rural, semi-tropical region that is composed of three provinces (Zarumilla, Tumbes and Contralmirante Villar), which are divided into 12 districts and then into villages. We have categorized farms in Tumbes as "small-scale" and "low-investment" since a combination of corralled and free-range swine-farming practices is found there. During the dry season, swine typically wander around the household or community at will, feeding on street garbage, weeds, or other refuse during the day. At night, the swine are maintained corralled on the farms. During the rainy season, the swine are typically kept on the farms at both day and night.

### Study participants

Because this is a qualitative study, a sample size calculation was not done. Instead, we used purposive sampling to literally seek out participants "with a purpose," that of being able to provide in-depth information about swine-associated interspecies interactions. We employed maximum variation purposive sampling, which aims to select participants who represent a broad range of possible variations in the topic of interest [26]. For this reason, we selected the two settings just described, confined pig farming in Chancay and low-investment pig farming in Tumbes. Additionally, within each setting, we sampled from a range of farms that varied according to the criteria that are most relevant to the realities of pig farming and possible interspecies interactions in the two contexts (Table 1). In Chancay, since all of the farms are located at different positions on the same hills above the ocean, we sampled from farms that are situated at different heights on those hills. In Tumbes, there are farms in all of the different villages, which are located at different distances from Tumbes. Therefore, we sampled from farms in 17 villages, which were classified as short, medium and far distance to Tumbes city. We also sampled from those that were close to the coast, with presence of wild birds, given that wild birds in Peru have been found to be reservoirs of IAVs [2]. We sampled participants until reaching a point of saturation, when new data no longer emerged. In Chancay, the research team worked with a veterinary technician "gatekeeper" to select participants. In Tumbes, the team benefited from on-going swine research work in the area and worked with the local research team to select participants. In total, 36 swine farmers participated in this part of the study.

**Table 1:** Small-scale swine farms observed in Chancay and Tumbes, by livestock practice and location within study site

	Swine farming with 10 or more swine		Swine farming with < 10 swine		Total farm- based observations	**Open location (street**, **plaza)**
	Mixed raising*	Only swine	Mixed raising	Only swine		
**Location in Tumbes**						
Village close to Tumbes city	1	1	1	1	4	1
Village medium distance to Tumbes city	1	1	1	1	4	1
Village far distance from Tumbes city	1	1	1	1	4	1
Close to coast, presence of wild birds	1	1	1	1	4	1
**Location in Chancay**						
High on hill	2	2	2	2	8	
Middle of hill	2	1	1	2	6	
Low on hill	1	2	2	1	6	
**TOTAL**	9	9	9	9	36	4

* Raising of swine with other domestic animals, including chickens, ducks, cows and sheep.

Additionally, the research team selected key informants to complement the information gathered in the observations. Seventeen individuals - nine farmers, one veterinarian, two veterinary technicians, three slaughterhouse workers, one agronomist, and one chicken vendor - were interviewed.

### Data collection activities

Direct observation was the primary data collection activity. Key-informant interviews were conducted to complement the information collected through observations.

#### Direct observation

In this data collection activity the researchers observed the actual events, behaviors, and practices occurring in the swine farms [26]. In total, 36 observations of farms were done: 16 in Tumbes and 20 in Chancay (Table 1). Each observation lasted an average of one hour, depending on the amount of activity in the farm. Observations were adapted to the local reality. In Chancay, the swine farmers generally do not live at their farms, but rather visit them twice a day-early morning and late afternoon-to feed the swine and to clean the pens. Therefore, observation in Chancay was carried out in the morning between 7 am and 11 am and in the afternoon between 3 pm and 5 pm. One morning observation was carried out at each of the Chancay farms and a second afternoon observation was carried out at eight farms. Afternoon observations were not carried out at each farm since the phenomena observed in the morning and afternoon were similar across farms, demonstrating "saturation" of data. In Tumbes, the majority of farmers keep their swine in "patios" or corrals adjacent to their homes. Since their contact with their animals is sporadic throughout the day, one observation was done at a random point during the day in each "home farm." Additionally, since many farmers allow their swine and other animals to roam freely through the community, one observation was carried out in the main plaza of four different villages in Tumbes.

We used an open-ended observation guide that was divided into four 15-minute segments, which allowed for more precise recording of the duration of interspecies interaction events. Observations were carried out by two of the authors (SM and MAR). Both observers were trained by AMB and then carried out observations of two of the same pig farms in Chancay in order to apply the lessons learned during training. Following these observations, SM, MAR and AMB met to review the two sets of observations line by line, discuss any differences, and establish guidelines to standardize observation practices.

Observers recorded data on the following: avian-swine interactions; human-avian interactions; and human-swine interactions. We did not limit the type of interactions to observe since the goal was to observe any and all interactions that emerged. We also noted the hygienic and sanitary conditions of the farms, as well as the farmers' hygiene practices: use or non-use of boots, gloves, and face masks, their hand-washing practices, and whether or not they touched their faces while on their farms.

#### Key-informant interviews

Key informants were interviewed by two of the authors (SM and MAR) using semi-structured interview guides with questions specific to their respective involvement with swine. For example, farmers answered questions that aimed to yield information on the amount of interaction they had with their swine, the time spent per day working on their farm, animal raising practices, farm and corral sanitation, and personal hygiene habits. All interviews were audio-recorded.

### Data analysis

The observations were compiled into word-processing software. Two of the authors (SM and AMB) then created a set of codes based on the key themes that emerged: avian-swine interactions (consumption of poultry mortality by swine; presence of wild birds, chickens or ducks inside swine pen; presence of wild birds, chickens or ducks on wall of swine pen); human-avian interactions (humans handling dead chickens; humans touching chickens or ducks; humans feeding chickens or ducks); and human-swine interactions (humans removing swine feces; humans feeding swine; humans providing veterinary care to swine; humans swatting or patting swine; humans picking up swine; swine sniffing or nuzzling humans). We also noted the hygienic and sanitary conditions of the farms, as well as the farmers' hygiene practices: use or non-use of boots, gloves, and face masks, their hand-washing practices, and whether or not they touched their faces while on their farms. ATLAS.ti software (Scientific Software Development GmbH, Berlin, Germany) was employed to apply these codes to the observations. Then, the data were synthesized into an Excel matrix and transferred into Stata 11.0 (StataCorp LP, College Station, TX). We generated the frequencies for each group and analyzed the differences between groups (confined farming in Chancay and low-investment farming in Tumbes) using Chi-square tests with Fisher's exact. The audio-recordings of the in-depth interviews were transcribed verbatim, and were analyzed in conjunction with the data from the observations. This method allowed the researchers to identify any similarities or discrepancies between the more subjective information from the in-depth interviews and the actual, observed swine-raising practices from the observations.

## Results

### Interspecies interactions

#### Avian-swine interactions

There were infrequent wild bird-swine interactions, despite the proximity of a protected avian sanctuary to the Chancay swine farms and the propinquity of the beach in both Tumbes and Chancay. In contrast, interspecies interactions between swine and poultry, such as ducks and chickens, were more common. The presence of these poultry species inside swine pens was similarly frequent in Chancay and Tumbes. Additionally, chickens and ducks were found perching on the walls of swine pens, although slightly more often in the corrals in Chancay than in Tumbes (Table 2).

**Table 2:** Interspecies interactions on small-scale swine farms in Chancay and Tumbes^a^

Avian-Swine Interactions	Chancay (N = 20) % (n)	Tumbes (N = 16) % (n)	p-value
Consumption of raw/cooked poultry mortality by swine	30.0% (6)	0% (0)	p = 0.024
Presence of chickens/ducks inside swine pen	35.0% (7)	37.5% (6)	p = 0.575
Presence of chickens/ducks on wall of swine pen	30.0% (6)	18.8% (3)	p = 0.353
**Human-Avian (chickens and ducks) Interactions**			
Humans handling dead chickens	30.0% (6)	0% (0)	p = 0.024
Humans touching chickens/ducks	10.0% (2)	0% (0)	p = 0.492
Humans feeding chickens/ducks	10.0% (2)	25.0% (4)	p = 0.374
**Human-Swine Interactions**			
Humans removing swine feces	95.0% (19)	12.5% (2)	p < 0.001
Humans feeding swine	80.0% (16)	87.5% (14)	p = 0.672
Humans providing veterinary care to swine	10.0% (2)	6.3% (1)	p = 1.000
Humans swatting/patting swine	80.0% (16)	68.8% (11)	p = 0.470
Average number of times per farm	3.3	1.3	
Humans picking up swine	30.0% (6)	6.3% (1)	p = 0.104
Average number of times per farm	3.0	1.0	
Swine nuzzling/sniffing humans	60% (12)	12.5% (2)	p = 0.006
Average number of times per farm	1.6	1.0	

^a ^All tests using Fisher's exact.

**Figure 1:**
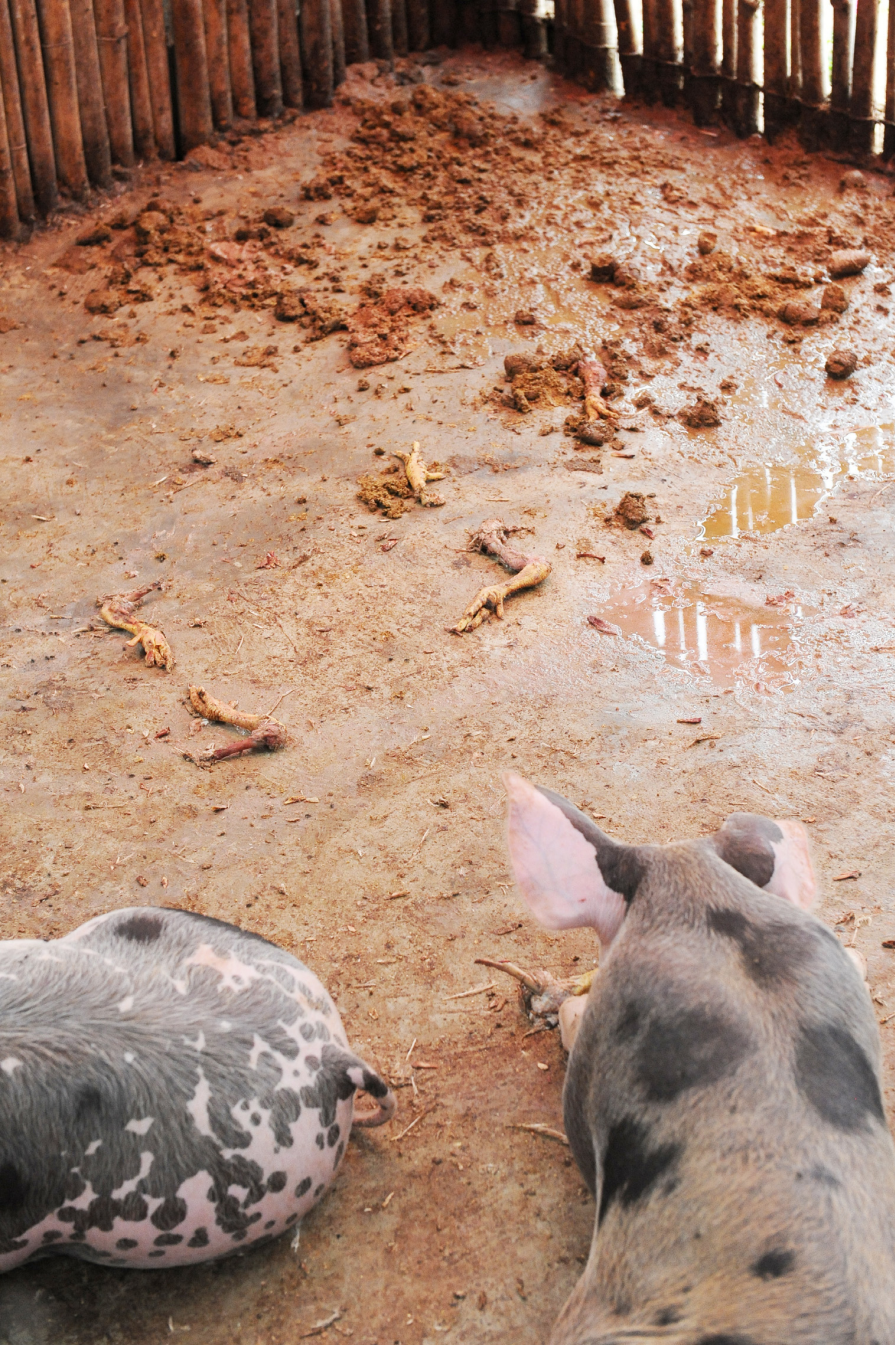
**Photograph of remnants of raw poultry mortality in a swine pen in Chancay**.

**Figure 2:**
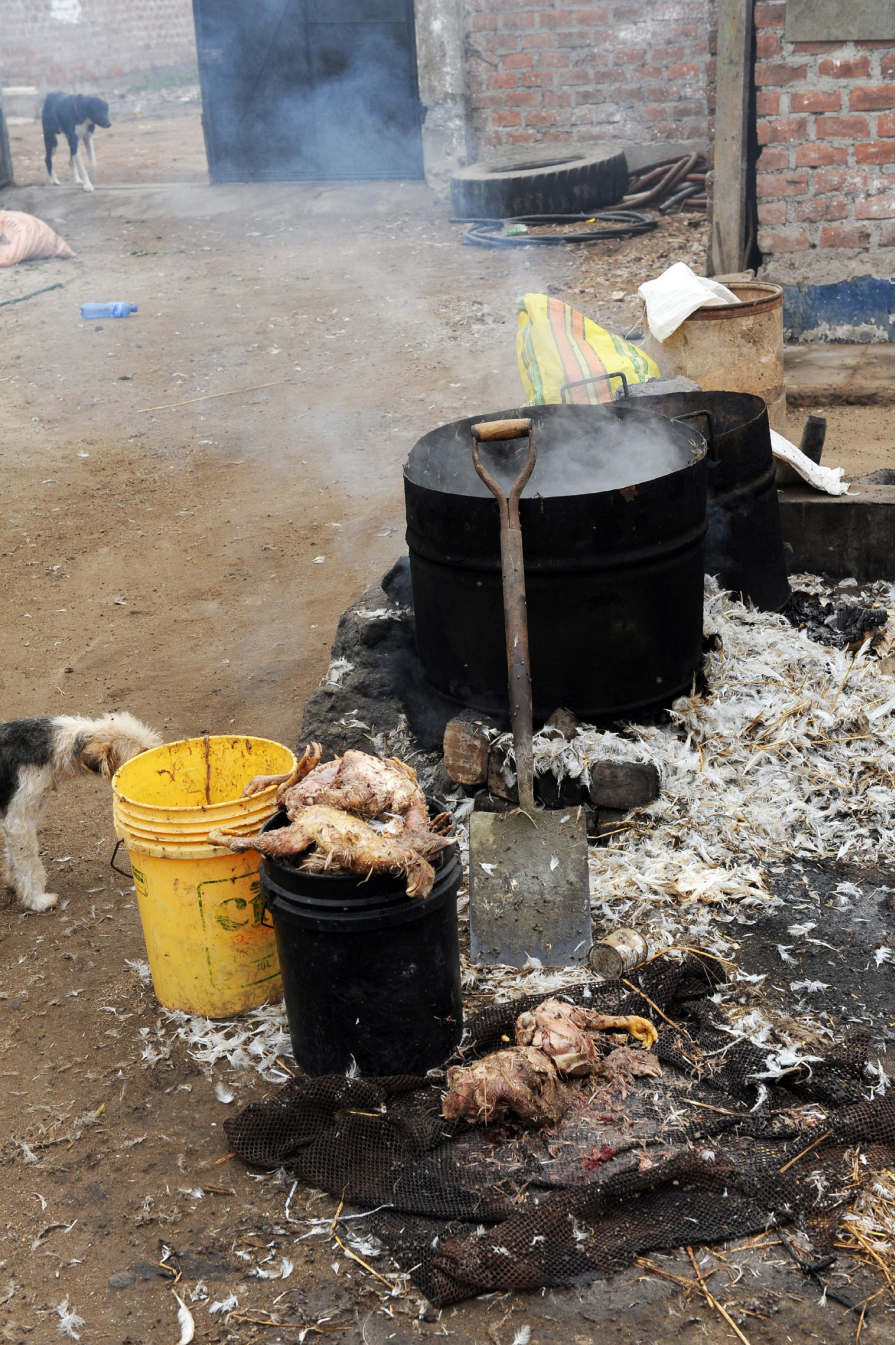
**Photograph of poultry mortality being boiled prior to being fed to swine in Chancay**.

An unexpected contact with avian species was that swine were fed poultry mortality in 6/20 of the farms in Chancay, either raw (n = 3, Figure 1) or cooked (n = 3, Figure 2). In Tumbes, no swine were observed consuming poultry mortality (Table 2). Poultry mortality refers to chickens that have not been slaughtered, but have died from disease or overcrowding. Because of this unexpected finding, farmers were questioned about the preparation of the poultry mortality. One farmer, who boiled the chicken for three hours prior to feeding it to her swine, said that her pigs would not eat chicken unless it was boiled. Another farmer, however, did not observe the same rejection of uncooked chicken. Therefore, this person did not boil the poultry mortality due to the time required and instead fed it without plucking and boiling.

In the in-depth interviews, farmers in Chancay noted that poultry mortality was an economical and nutritionally beneficial supplement to their swine's diet. A farmer related that she gave her swine commercial feed until they were two months old, switching thereafter to a stew-like mixture of poultry mortality and household leftovers. The reason for the switch was to save money spent on feed. Two other farmers specifically stated that feeding poultry mortality to their swine allowed them to save on feeding costs. A fourth farmer said that she fed chicken blood to her swine starting at two and a half months and whole poultry mortality at three months. Key-informant farmers indicated that the sources of poultry mortality were three local chicken vendors. Though it is illegal, personnel from some industrialized poultry farms in the area have arrangements in which they clandestinely sell their poultry mortality to local chicken vendors who in turn sell the products to swine farmers. One chicken vendor key-informant estimated that he sold 100 to 150 poultry mortality per week to swine farmers.

#### Human-avian interactions

Farmers primarily interacted with their poultry during their feedings, which usually consisted of throwing grain to a small flock ranging in size from two to six birds. The feeding of poultry was observed at least once in 25.0 percent of farms in Tumbes and in only 10.0 percent of farms in Chancay. However, the average number of poultry-feeding instances per hour observed was higher in Chancay (3.5 times) than Tumbes (1 time). Additionally, poultry touching by a human was noted in 10.0 percent of the farms in Chancay at an average of one time per hour observation. In Tumbes, there was no such record of human-avian interaction.

Farmers in 6/20 sites in Chancay handled either raw or cooked poultry mortality when feeding it to their swine. During one observation in Chancay, a farmer opened a large feedbag full of poultry mortality -whole and with feathers- and manually threw it into three different swine pens. This person was not wearing any hand protection equipment.

#### Human-swine interactions

Feeding was the most common human-swine interaction in both study settings. In order to feed their swine, farmers in Chancay entered the actual pens to pour food into concrete or rubber tire troughs, and either remained inside to clean or entered an adjacent pen to feed other swine. This feeding method contrasted sharply with that observed in Tumbes: farmers there dumped the swine's food into the troughs by leaning over the pens' gates or walls. They did not enter or remain inside the pen for even short periods of time, which, consequently, curbed their interactions with swine. Similarly, the removal of swine manure from pens was very frequent (95%) in Chancay, while only 12.5 percent of farmers were observed removing manure in Tumbes (Table 2).

The provision of veterinary care was observed on 10.0 percent of the farms in Chancay and on 6.3 percent in Tumbes. In Chancay, a farmer during the same observation period used the same syringe to inject medication into two different piglets. During another observation, a farmer in Chancay rubbed salve on the wounds of newly castrated piglets. A farmer in Tumbes used an unidentified liquid on a swine to treat what appeared to be scabies.

In both Chancay and Tumbes, the swatting or patting of swine was observed to be a mechanism for urging the swine to move out of the way for cleaning or feeding purposes. Farmers typically used their bare hands or other tools, such as a stick or a spade, when swatting or patting the head or the side of their swine. In Chancay, 80.0 percent of farmers swatted or patted their swine at an average of 3.3 swats or pats per hour. Swatting and patting by farmers was similarly present in 68.8 percent of Tumbes farmers, although it was less frequent, at 1.3 swats or pats per hour. More direct contact occurred when farmers picked up their pigs, an activity that was more frequent in Chancay (30.0%) than in Tumbes (6.3%). On average, swine were picked up three times per farm in Chancay and once in Tumbes.

Another contact interaction occurred when pigs nuzzled or sniffed the farmer. This contact occurred more often in Chancay (60.0%, 1.6 times per observation) than in Tumbes (12.5%, 1.0 time per observation), which is likely due to the fact that famers in Chancay spent an appreciably greater time in the swine pens.

#### Observations at plazas

Four observations were carried out in open plazas in Tumbes, in areas where scavenging swine were known to frequent. During these observations, the swine did not have interspecies contacts with birds, dogs or humans.

### Hygiene practices

#### Boots, gloves and face masks

All farmers in Chancay wore some sort of footwear and the majority (60.0%) wore boots. By contrast, in Tumbes, 25.0 percent of farmers were barefoot while interacting with their swine. The vast majority (62.5%) used rubber or plastic thong sandals (flip-flops), 12.5 percent wore closed-toed shoes (not boots), and no one wore boots. Hand protection in the form of gloves was observed for only one farmer participant across both settings. Finally, no farmers wore face masks during the observations (Table 3).

**Table 3:** Hygiene practices among farmers on small-scale swine farms in Chancay and Tumbes^a^

Protective Clothing	Chancay (N = 20) % (n)	Tumbes (N = 16) % (n)	p-value
***Footwear***			p < 0.001
Barefoot	0% (0)	25.0% (4)	
Flip-flops	40.0% (8)	62.5% (10)	
Closed-toed, not boots	0% (0)	12.5% (2)	
Boots	60.0% (12)	0% (0)	
***Handwear***			p = 1.000
Bare hands	95.0% (19)	100.0% (16)	
Gloves	5.0% (1)	0% (0)	
***Face protection***			p = 1.000
Mask	0% (0)	0% (0)	
**Hand-Washing Practices**	**% (n/N)**	**% (n/N)**	p = 0.731
No washing	45.0% (9/20)	56.3% (9/16)	
Water only	25.0% (5/20)	25.0% (4/16)	
Water and soap	20.0% (4/20)	6.2% (1/16)	
Water and detergent	10.0% (2/20)	12.5% (2/16)	
**Face Touching**	**% (n)**	**n/a**	n/a
No face touching	55.0% (11/20)	n/a	
Touched forehead	44.4% (4/9)	n/a	
Touched nose	55.6% (5/9)	n/a	
Touched mouth	0% (0)	n/a	

^a ^All tests using Fisher's exact.

#### Hand-washing

Forty-five percent of farmers in Chancay and 56.3 percent of farmers in Tumbes did not wash their hands at any point while working in their farms. Rinsing of hands with non-running water from large drums was the most common practice in both sites. The use of soap or detergent was observed in 25.0 percent of farms (Table 3). Among farmers who washed or rinsed their hands, only one farmer in Chancay did so twice, although without soap or detergent.

#### Face touching

We did not observe this type of practice in Tumbes. In Chancay, the majority of farmers did not touch their faces while working with their swine. However, among those who touched their faces (n = 9), more than half (55.6%) had hand-nose contact and 44.4 percent touched their foreheads.

## Discussion

Results from this study revealed that interspecies interactions were common to both study sites, though there was a higher frequency of interactions in Chancay, especially avian-swine and human-swine contact. An unexpected finding was the practice among Chancay farmers of feeding poultry mortality to swine, which may have important implications for interspecies influenza transmission.

One possible limitation of this study is its sample size. However, a small sample size was important for observing the issues of interest in a uniform manner and in great detail. Additionally, in order to observe possible differences across the different types of swine raising in Peru, two distinct study sites were selected for comparison. These sites provided a view of different swine-raising practices, as well as the risks these practices pose for interspecies influenza transmission. Furthermore, the authors acknowledge that the results of this study are not intended for broad interpretation; rather, they provide a solid foundation that further, expanded studies may build on. The qualitative tools employed to examine interspecies interactions may be considered as having some limitations. For example, direct, non-participant observation, the principal methodology utilized, can be subject to observer bias [26]. To limit this bias, observers were trained in how and what to observe, with a focus on what types of expected and unexpected interactions could be considered meaningful. This open-ended approach to observation was critical to our specific objective of observing all types of human-swine-avian interactions. It allowed the research team to pursue all relevant information that emerged and thus uncover interspecies contacts not previously anticipated through literature review. Another potential limitation with direct observation is that subject behavior is sometimes modified due to the presence of the researcher [26]. The research team explained to the farmers that they were present to observe the day-to-day activities on their farms and that they did not need to do anything differently because of their presence. The study findings seem to support that this approach worked, and that behavioral modifications due to our observations did not happen.

Despite these limitations, this study has several important findings.

### Avian-swine interactions

It has been well documented that the intermingling of swine and fowl in the same pen violates good biosecurity practices and increases the risk of interspecies influenza transmission [25,27,28]. The intermingling of species was noted with great frequency in both Chancay and in Tumbes: ducks and chickens were observed both roosting on swine-pen walls and roaming freely inside the pens.

A critical breach of biosecurity practices was the feeding of raw poultry mortality to swine, which occurred on 15 percent of farms in Chancay. This practice reveals a potential mechanism for interspecies contamination with pathogens of potential public health significance. Because IAVs can survive in temperatures as high as 72.2°C, these events pose significant risk of influenza transmission [29]. Although IAV H1N1 has not been previously reported in poultry in Peru, the environmental interaction of swine with poultry mortality and the feeding of poultry mortality to swine is a mechanism by which other pathogens including IAVs may be unnaturally introduced into swine. It is important to note here that dogs in South Korea were infected by avian H3N2 influenza virus, most likely from being fed infected poultry byproducts [30]. A similar event was the cause of Bovine Spongiform Encephalopathy where improperly rendered sheep were fed to cattle [31]. Feeding unprocessed or improperly processed carcasses can also result in the transmission of other pathogens-such as salmonella and campylobacter-that can infect both swine and humans [32].

### Human-avian interactions

This unexpected observation of the feeding of poultry mortality to swine was unique to Chancay, an area with a large number of industrialized poultry farms in close proximity to the small-scale swine farms included in this study. These small-scale farms, which are owned by swine farmers with limited income, represent a potential market for the poultry mortality byproducts of the much more affluent industrialized farms. This hazardous practice has also been reported in other parts of the world that have marked economical disparities, where swine farmers of lower socio-economic status use farm mortality from large, industrialized poultry operations [33]. The risk of interspecies influenza transmission from poultry to swine to humans may therefore be greater when such socio-economic gaps exist. This phenomenon contrasts with cysticercosis, where the disease is highly associated with a general lack of economic resources and proper sanitation in the area [34]. It is also important to highlight findings from Vietnam, where Dinh et al. (2006) observed that handling of dead or sick poultry in an IAV-affected area was a risk factor for human infection [35].

### Human-swine interactions

The swine farms in Chancay and Tumbes shared many similarities with swine farms in other developing countries. Like a number of farms in Vietnam, Laos, and India, Chancay swine farms were characterized as small-scale and confined, housing 20 or fewer sows in permanent structures [25,36-38]. In Chancay, farmers generally spent an average of three to five intensive hours working in their corrals, performing tasks including feeding swine twice a day, cleaning pens, and administering veterinary care. These tasks necessitated and often provoked interspecies interactions, and paralleled-with the exception of pen cleaning-the interspecies interactions on other small-scale, intensive farms in the developing world [25,36-38]. Increased time spent in a swine corral and close contact with the animal yields greater risk of pathogen exposure [39].

While small in scale like farms in Chancay, farms in Tumbes were significantly less developed and housed ten or fewer swine, a model similar to small-scale swine farms in parts of Africa [25,40,41]. Tumbes swine farmers operated what the Food and Agriculture Organization of the United Nations (FAO) et al. (2010) referred to as a "low-investment, semi-scavenging production system" [25]. These low-investment systems are generally favored by swine farmers with few resources or by those who raise swine for a secondary income. Consistent with findings from Nigeria and Kenya, swine in Tumbes were confined to small areas, such as yards, paddocks or basic shelters, which were haphazardly constructed of locally procured material and which allowed swine to enter and exit easily [40,41]. Additionally, farmers in Tumbes spent little time with their swine. In the vast majority of cases, Tumbes swine farmers limited their interactions to twice-daily feedings of household leftovers thrown into troughs without farmers even needing to enter their farms. Such brief human-swine interactions were also observed on small-scale, low-investment farms in Nigeria, Kenya, and Madagascar [40-42].

Irrespective of the location, farmers in both Chancay and Tumbes closely interacted with their swine in the provision of veterinary treatment. Farmer-administered veterinary care, observed in a small number of the farms in Chancay and Tumbes, is a practice that has been recorded on small-scale swine farms in northwest India and in northern Vietnam [36,43]. Treatments observed in this study included vaccinating piglets and topical care. Farmers in both sites noted that veterinarians were called upon in serious cases only, which is consistent with the findings of Kumarsean et al. (2009) and Lemke et al. (2006). Providing veterinary care required farmers to be in close contact with swine and thus increased the possibility of being exposed to influenza-infected aerosols, feces or fluids [44]. Conversely, if the farmers themselves were ill with influenza, their swine would also be at risk for infection.

### Hygiene practices

Observational data collected on hygiene practices showed that farmers in Chancay and Tumbes had little to no biosecurity measures in place, a finding that is consistent with earlier reports from Asian and African countries [38,42]. Hand-washing was performed slightly more often in Chancay than in Tumbes. In both sites, the majority of swine farmers were not observed washing their hands after interacting with swine or fowl. Among farmers who cleaned their hands after tending to their swine, about half only rinsed their hands with water. This suboptimal practice was also previously described in similar settings in other developing countries [36,45]. The remaining half of farmers that practiced hand washing used water and either soap or detergent, although soap was more common in Chancay and detergent was more common in Tumbes. Detergents, soap and other disinfectants can kill the influenza virus and hand washing that includes one of these products can help control interspecies influenza transmission [46]. Thus, education campaigns may be needed to emphasize the importance of proper hand-washing and correct the incorrect perception that rinsing hands with water is protective.

The use of protective clothing has been demonstrated to lower the risk of interspecies influenza transmission [47-49]. However, utilization of such clothing was lacking in Chancay and in Tumbes. Indeed, flip-flops were observed in the vast majority of swine farms in Tumbes and on a significant portion of farms in Chancay. Gloves were almost never used; only one farmer across both sites wore gloves when interacting with her swine. Similarly, face masks were never used. Without proper protective clothing such as rubber boots, gloves and face masks, humans are more likely to come into contact with IAVs though pathogenic animal secretions and feces [36].

Finally, it is important to note that these study findings may be relevant to the transmission of other pathogens. This could include campylobacter and toxoplasma, which - for example - are prevalent in chickens in Peru [50-52], as in other settings.

## Conclusions

Recognizing that small-scale swine farms located in areas of industrialized poultry farms constitute a public health concern may be key in preventing interspecies influenza epidemics. A two-pronged approach to prevention carried out jointly by local public health authorities, veterinarians and agricultural officials is important. First, they should provide educational activities for swine farmers that emphasize the health and economic benefits of sound hygiene and biosecurity practices. Hygiene education should center on the importance of soap or detergent in hand washing and promote the use of specific work clothing (including gloves and boots). Biosecurity education should encourage the separation of species and communicate the dangers of feeding raw chicken to swine, in addition to providing guidelines about how to appropriately boil chicken. Second, they should establish guidelines and educate poultry farmers about alternative approaches for processing poultry mortality. Approaches include rendering, which can result in an affordable source of protein meal that farmers can feed to their chickens, or composting, a low-cost strategy that can result in a fertilizer byproduct that can generate additional income for the farmers. Finally, virological and serological surveillance for influenza viruses will also be critical for these human and animal populations.

## Competing interests

The authors declare that they have no competing interests.

## Authors' contributions

SM, CSA, RHG and AMB conceived and designed this research. SM and MAR performed the field work with support from CSA, RHG, VA, AEG and AMB. SM carried out the data analysis with support from CSA, RHG and AMB. All authors contributed to the interpretation of the data and the preparation of the manuscript.

## Pre-publication history

The pre-publication history for this paper can be accessed here:

http://www.biomedcentral.com/1471-2334/12/58/prepub
